# A riddle, wrapped in a mystery, inside an enigma: How semantic black boxes and opaque artificial intelligence confuse medical decision‐making

**DOI:** 10.1111/bioe.12924

**Published:** 2021-08-10

**Authors:** Robin Pierce, Sigrid Sterckx, Wim Van Biesen

**Affiliations:** ^1^ Tilburg Institute for Law, Markets, Technology, and Society Tilburg Law School Tilburg The Netherlands; ^2^ Bioethics Institute Ghent Ghent University Ghent Belgium; ^3^ Consortium for Justifiable Digital Healthcare University Hospital Ghent Ghent Belgium

**Keywords:** algorithms, clinical care, decision support, e‐alerts, medical AI, medical semantics

## Abstract

The use of artificial intelligence (AI) in healthcare comes with opportunities but also numerous challenges. A specific challenge that remains underexplored is the lack of clear and distinct definitions of the concepts used in and/or produced by these algorithms, and how their real world meaning is translated into machine language and vice versa, how their output is understood by the end user. This “semantic” black box adds to the “mathematical” black box present in many AI systems in which the underlying “reasoning” process is often opaque. In this way, whereas it is often claimed that the use of AI in medical applications will deliver “objective” information, the true relevance or meaning to the end‐user is frequently obscured. This is highly problematic as AI devices are used not only for diagnostic and decision support by healthcare professionals, but also can be used to deliver information to patients, for example to create visual aids for use in shared decision‐making. This paper provides an examination of the range and extent of this problem and its implications, on the basis of cases from the field of intensive care nephrology. We explore how the problematic terminology used in human communication about the detection, diagnosis, treatment, and prognosis of concepts of intensive care nephrology becomes a much more complicated affair when deployed in the form of algorithmic automation, with implications extending throughout clinical care, affecting norms and practices long considered fundamental to good clinical care.

## 1 INTRODUCTION

The introduction of artificial intelligence (AI) into healthcare to improve outcomes holds out considerable promise. The possibility of automated processes that can facilitate preventive care, early detection, and more effective treatment is worthy of careful and serious consideration. However, with medical innovation, the purported benefits are almost invariably accompanied by drawbacks, challenges, and unanticipated consequences. A specific challenge that remains underexplored in the emerging literature on the ethics of the use of AI in healthcare is *the lack of clear and distinct definitions of the concepts used to develop the algorithms*, and how their real world meaning, when translated into machine language and the subsequent interpretation of their output by physicians, can *alter the outcomes* in clinically significant ways. Such semantic confusion can arise as a result of different mechanisms: (a) the assumptions and metrics used to translate the definition of a concept into an algorithm are unclear (e.g., the concept of acute kidney injury [AKI] can be translated into an algorithm in many different ways, each referring to distinct clinical conditions); (b) dichotomization or categorization of continuous variables into classes; (c) framing—qualitative rather than quantitative output, for example, using terms like “better” or “more”; (d) mixing up different conceptual constructs (e.g., the comparison of “early” versus “late” start of renal replacement therapy, where words with a time connotation are used to describe a decision in fact based on renal function level. Of note, in this case, there is also framing as “late” has a negative connotation per se).

The “semantic” confusion present in many medical AI systems can have considerable implications and consequences for clinical care also because the underlying “reasoning” process is frequently opaque, essentially converting the process and its output into semantic as well as mathematical black boxes. Rather than being more objective or precise, the true relevance or meaning of an AI output to the end‐user is frequently obscure. This problem also exists in the “normal” medical decision‐making process, but is *further aggravated and multiplied by the use of algorithms*. Indeed, whereas in normal medical decision‐making, each physician will decide individually using her own judgment, and the impact of mistakes is thus limited, implementation of one single algorithm can corrupt the decision process for multiple users, all of whom will make the same incorrect interpretation. AI devices are used not only for diagnostic support by healthcare professionals, but also to inform treatment decisions to patients, and thus affect the practice of shared decision‐making, informed consent, and the doctor–patient relationship, in general. This paper explores the ethical implications of this phenomenon through the lens of cases from intensive care nephrology where algorithms are being developed to detect, diagnose, and inform treatment decisions regarding conditions such as AKI.

## 2 BACKGROUND

### 2.1 Automated monitoring and detection

“Narrow” AI applications, operating within strictly delineated settings and aimed at solving well‐defined problems such as classification and prediction tasks for diagnosis or prognosis,^1^ can be a breakthrough for medicine.^2^ Following a nearly 70‐year trajectory of interest in machine learning, AI appears to have turned a corner with the transition from “knowledge‐engineering” to a more optimized data‐driven technology predicting and deriving new insights.^3^ However, it is still uncertain to what extent AI will prove useful in actual clinical practice, and clinical embedding and ability to ascertain clinical efficacy remain challenging.

There is expanding interest in AI‐based automated alerts and prediction. Trained on vast amounts of data from registries or electronic health records (EHRs), AI holds out the promise to enhance timely detection and diagnosis, and facilitate earlier treatment. AI based automated systems that calculate the crossing of “thresholds” for medical conditions or generate predictions of mortality rates have tremendous appeal in over‐burdened and under‐staffed healthcare settings and in low resource conditions. However, such systems often lack transparency about the exact nature of the medical construct that is identified.

Researchers have long acknowledged the complexity of the promises and limitations of the use of computer aids in medicine, particularly for diagnosis. In 1959, Ledley and Lusted declared the practical necessity of standardization of nomenclature (and test interpretations), observing that “while the computer can be made to recognize different words as denoting the same idea, it obviously cannot distinguish between different ideas denoted by the same word.”^4^ The multiple levels of complexity that are involved in transferring language to automated digital systems should not be underestimated. Some of these challenges have been acknowledged and addressed to varying degrees, while others remain unrecognized or under‐explored.

One problem that has been neglected until now regards the lack of exact and transparent definitions of many clinical, radiological, and biochemical constructs within AI algorithms. In cases where a sharp definition *does* exist, problems can emerge with how the terms are *translated* into the mathematical language of algorithms. Without clear and traceable definitions, users cannot discern the exact meaning of the labels produced by the algorithm. For many medical conditions, the absence of strict and clear definitions is one of the many wrinkles that are accepted and tolerated as part of routine medical practice.^5^ The problem of semantic confusion in machine learning and AI is different from other digital formats because most learning systems “upgrade” themselves as new cases come in. At a certain moment, a self‐perpetuating event arises in which the computer just becomes more and more confident of the diagnosis made based on new cases when a correct diagnosis was made based on what was learned before. This problem has so far mainly been recognized and deemed relevant for epidemiology^6^ and randomized clinical studies. However, with the advent of EHRs, algorithms to automatically diagnose and predict medical conditions are increasingly being developed and implemented in the care of patients.^7^ The use of automated risk prediction models in the EHRs of ICUs for early recognition of different acute medical conditions, such as AKI,^8^ brings the problem of the lack of accurate and precise definitions of medical constructs to a different level. Indeed, whereas the attention and action span of an individual physician is limited to some patients and at a limited frequency, an algorithm can be used to supervise thousands of patients in hundreds of hospitals on an ongoing basis. This alters the total picture of patient monitoring yet the meaning of the reported condition remains obscure.

### 2.2 The semantic black box in the case of AKI

A universally accepted standardized definition exists for AKI, based on the so‐called Kidney Disease Improving Global Outcome (KDIGO) criteria. However, in reality, AKI remains a largely opaque medical construct. This opacity is further exacerbated by algorithmic automation. Essential components of algorithms may not be available within local data sets according to uniform definitions. This may negatively impact clinical usefulness and relevance of these systems and threaten performance.^9^ Further, lack of harmonization and inter‐operability may exacerbate concerns of poor generalizability from training to validation settings due to center‐related differences in patient case mix and clinical practice. The emergence of algorithmic software purporting to operationalize the KDIGO criteria thus opens the door for a widely diverging range of quality in the accuracy, effectiveness, and usefulness of the technology if deployed in the clinical setting. Using marginally different instructions to operationalize computer algorithms for AKI diagnosis in critically ill patients, even when still compliant with the uniform and standardized KDIGO definition, will have a substantial impact on the *incidence* of AKI and its *association with short‐term mortality*. Moreover, these semantically rooted challenges compromise the prognostic value of algorithms meant to predict ICU mortality of AKI patients.

The semantic black box in the case of AKI is particularly interesting and illustrative because the phenomenon does not necessarily stem from the use of AI per se, but is inflated by its use. Indeed, the semantic black box is already present in the nonautomated KDIGO criteria. But the automation of this semantic confusion and the proliferation of varying quality of algorithms and widespread uptake by insufficiently trained personnel can be expected to lead to a Pandora's Box of undesirable clinical sequelae. The absence of adequate and appropriate regulation and governance of algorithmic systems and a means of ensuring that terms translated into models carry a more precise meaning will have considerable consequences when deployed at scale. In the next section, we will explore the specific nature of the definitional conflation of AKI and, thereafter, examine the multiple ways that translation of this semantic black box exacerbates the opacity and carries significant implications for clinical care.

## 3 THE BLACK BOX OF MEDICAL CONSTRUCTS

Medical practice is steeped in jargon, acronyms, and amorphous terms, leading to confusing and misleading communication. Nephrology is no exception. For example, the question of whether to administer dialysis “early or late” is impossibly vague, setting up a frame that can influence treatment decisions, but doing so on a highly imprecise basis. The use of the terms “early” and “late” creates the impression of *timing*, which in reality is not present. The concept of starting dialysis is based on serum levels of toxins that accumulate in the blood of patients whose kidney function progressively fails, not on a time dimension. Moreover, these terms are not neutral, as “late” carries a negative connotation. The terminology used is thus likely to influence a patient's decision, but conveys remarkably little relevant information. Definitions and clinical diagnoses potentially pose a considerably greater problem. Amorphous terminology may be sufficient in some settings to communicate (e.g., “hanging in” conveying that the patient is not dead yet), but the precision with which a particular term, characterization, or diagnostic label identifies the status, condition, likelihood of survival or other clinically relevant attributes, can sometimes be seriously problematic.

The complexities, variations, and alternative interpretations contained in (diagnostic or prognostic) categories give rise to concerns and dilemmas for even the most skilled clinical practitioners. For example, a paper on the management of hyponatraemia (too low salt level), may state that this is a frequent condition with a high mortality. However, the reference to underpin that the condition is frequent refers to a very subtle degree of hyponatraemia in ambulatory patients, whereas the reference on the high mortality refers to very pronounced hyponatraemia in patients in intensive care. The ensuing conclusion is thus at best misleading, as the construct hyponatraemia clearly has a different implication in both contexts. When data scientists try to translate terms, definitions, and diagnoses that are amorphous and subject to considerable variation in usage into computer language, what was complex and conflated in human communication becomes even more so due to the opacity of the processes and the training data, which may or may not be relevant for the patient being treated.

### 3.1 Detecting AKI: Criteria, communication, and confusion

The clinical practice of using the same term to describe a range of conditions may be efficient for some practical reasons but can become problematic in cases requiring well‐chosen clinical interventions. In the literature published before the year 2000, the term “acute kidney failure” was used. However, the term lacked sufficient precision. Accordingly, there was much confusion regarding the interpretation of for example, epidemiological data, or the effectiveness of therapeutic interventions, as it was difficult to find out what exactly was understood by “acute renal failure.”^10^ AKI as defined by the widely accepted KDIGO criteria may seem to be more precise than “kidney failure,” but in fact, this is not necessarily the case. Two measures are essential for an AKI diagnosis—creatinine (SCrea) and urinary output (UO). However, the manner and timing of these measurements can vary considerably. A systematic review has revealed that in different publications on automated detection of AKI, 44 different interpretations have been used.^11^ This feature becomes particularly important when assembling the training data for an algorithm that will aim to detect AKI. That is, when developers operationalize SCrea and UO criteria into an algorithm to automatically identify patients with AKI, the result is a *high variability* of both AKI incidence and its prognostic value for ICU mortality. Yet all interpretations remain strictly in line with the standardized KDIGO‐AKI definition, and differences are merely a result of decisions made when operationalizing the criteria to the algorithm. In addition, it is often claimed that KDIGO criteria are used, but, in reality, only a truncated version is used. This is because *laboratory* data such as serum creatinine are easily available in electronic databases, whereas *clinical* data such as UO are much more difficult to obtain. In reality, 95% of studies on automated AKI detection only used SCrea and not the UO criteria. As a result, most of these seemingly objective and robust automated AKI detection systems are profoundly opaque black boxes that can generate a positive or negative AKI label for a particular patient with significantly different clinical consequences. These decisions are rarely available to the end‐user, so she also cannot know exactly *which* type of AKI is detected. In this case, the “black box” is not only *mathematical* (the end‐user does not know how input leads to the output mathematically), or *logical* (which reasoning process is followed by the AI), but also *semantic* (the output can, despite appearing unequivocal, signify a variety of conditions).

### 3.2 Multiple metrics of KDIGO: A history

Building on decades of scientific and clinical research, the KDIGO AKI definition^12^ emerged in 2012 as a an integration of previous definitions^13^ as an intentionally broadly constructed definition of acute kidney damage, allowing for, among other things, a wide range of tests, procedures, and interventions to be classified and reimbursed accordingly. As a result, AKI according to KDIGO criteria can include measurements of some of the following five baseline metrics for creatinine: (a) obtained before hospitalization; (b) a back‐calculation; (c) measurement from ICU blood; (d) the lowest pre‐ICU measurement from current hospitalization; and (e) immediate pre‐ICU measurement. Additionally, UO is also informative, but is measured in one of two ways: (a) measured for a total of 12 hr; or (b) measured each hour for 12 hr.

When these diverse metrics related to measuring kidney function, *but indicative of different problems*, are bundled into a single definition, *the medical construct of AKI becomes, itself, a black box*. Operationalization into an algorithmic model essentially places the black box of the medical construct within another, faster, algorithmically driven automated black box that generates outputs indicating that some threshold for kidney dysfunction has been reached. As a set of measures of convenience, for example, in hospital measurement versus available historical levels, the resulting calculations are not and cannot truly be commensurate. The clinical encounter with a knowledgeable nephrologist familiar with the patient and her clinical history most likely will yield a better understanding of how to interpret the measurements within the general context. Once automated, knowledge arising from the clinical encounter with this particular patient falls away, but the automated device will still produce a result that the clinician has difficulty interpreting but for which both rejection and acceptance have legal and ethical consequences. In addition, if the clinician would have to check all results for their credibility, the whole purpose of having the automated devices becomes pointless. While massive big data may fill some of the void, the scale and exacerbated opacity of an AI‐generated label of AKI renders the output less clinically helpful in some very disturbing ways.

### 3.3 Clinical consequences

Although formal medical practice has lived with imperfection since its inception, the automation of these imperfections can have multiple small cumulative impacts affecting life or death. The possible reach of an automated flawed medical construct is long, with consequences for patient care and the practice of medicine, including the patient–doctor relationship, professional and moral responsibility, and even what constitutes medical knowledge.

Among the foreseeable general impacts of automated AKI detection is a tendency toward over‐diagnosis. Given that the KDIGO criteria are so broad and all‐encompassing and, even in human hands, result in a widely acknowledged over‐diagnosis of AKI, it is important to note that the fact of automation itself and the use of EHRs to inform e‐alerts amplifies this by the speed, efficiency, and scale that AI‐driven systems can achieve. So, while the clumsy AKI label generates a certain number of false positives on its own, when automated, that number increases, conceivably exponentially. The consequences of false positives can be quite serious as the management of this patient may change substantially, for example by additional volume loading or by reducing dose or simply withholding certain medications that are necessary but potentially toxic to the kidneys. As such, an incorrect AKI label could worsen the patient's condition.

AI based e‐alerts to facilitate treatment of kidney patients have considerable appeal. E‐alerts are used to monitor patients in ICU for detection of a change of condition that meets the modeled criteria for AKI and to signal this to clinical staff. It is easy to understand the appeal of this monitoring technology for such a critical signal. In principle, over‐burdened care institutions can provide better and more timely monitoring for acute changes for more patients with fewer highly skilled staff. This is a deceptively problematic aspect of what is purported to be a benefit of the algorithmic e‐alert. First, while an e‐alert signals that a patient has AKI, it does not tell the attending staff what the *cause* is or which *intervention* is required to effectively respond to a particular patient's condition. Thus, while the e‐alert seems a significant advance in timely detection of AKI, it can lead to many negative consequences due to the automated flawed medical construct, ranging from over‐diagnosis and insufficient information for attending staff to identify the most appropriate intervention to alert fatigue.

Even more concerning are the possible clinical consequences of false positives—the triggering of an AKI e‐alert when the patient does not actually have AKI. This could be because of a mismatch between the patient's metrics and those on which the algorithm was trained, stemming in no small part from variable interpretations of the KDIGO criteria that have been applied to the training data. Furthermore, the e‐alert is of only limited benefit if it merely signals a *detection* of AKI, without any explanation of why a particular patient has triggered that alert. For example, the most common reason to get an e‐alert is oliguria (decreased urine production). The most frequent treatment is fluid. However, if there is another problem that has caused the decrease in urine production, a fluid treatment can have devastating consequences. Moreover, because of the dichotomous nature of AI outputs—either AKI or not‐AKI—the conditions on the continuum designating “at risk” are essentially being ignored, thus resulting in a missed opportunity for personalized, adapted intervention.

## 4 “MEDICAL KNOWLEDGE”: WHAT DO WE REALLY KNOW?

There is also impact on what we generally consider to be “medical knowledge.” Indeed, if the diagnosis (AKI) represents a collection of metrics known to be related to the onset of AKI, but does not distinguish between baseline measurements in generating the outputs (assessment) and cannot identify the cause of AKI in a specific patient, this calls into question what exactly is known by the application of the KDIGO criteria in the first place, and by an algorithmic model operationalized to calculate thresholds based on the KDIGO criteria in the second.

With implementation of nonautomated KDIGO criteria, the medical knowledge that is produced by evaluating the results of one or more of the criteria described above (different times of measurements of creatinine and urine output levels [UO]) allows a physician to know which criteria were applied and which were not. For example, the UO measure has proven to be much more informative of prognosis than the creatinine levels. Thus, a nephrologist relying on a prognosis from KDIGO criteria can presumably check whether UO levels were included in order to assess prognostic value of the AKI diagnosis or to request that those measurements be taken. The operationalization of KDIGO in an automated system renders the calculation of the constituent criteria (in both content and degree) largely a mystery.

This also leads to *generalizability* problems. Given that if it is unknown on what the AKI e‐alert is based, it is difficult to say what the data that were generated from inclusion of a particular AKI patient group (and how those data were obtained) signify for other patients. In this way, in the case of deep learning, the black box, as both collector and generator of data, compounds the opacity that may result in incrementally more accurate alerts but also increasingly obscures what the e‐alert actually means. Moreover, the general practice of medicine across locations employs different definitions in different studies. Consequently, automating the collective “knowledge” resulting from these studies can generate confusing outcomes because the definition, used to determine eligibility and diagnostic status, as well as the implementation of the criteria across the studies, were different.

The amplified imprecision brought about as part of the translation to algorithmically‐driven automated alerts also amplifies the causation problem. As with randomized controlled trials (RCTs), the knowledge gained largely regards correlation. Yet, with RCTs, the results allow for an understanding of specific correlations of patient subsets that contribute to the overall correlative conclusions. No such illumination of specific correlations regarding the patient's specific characteristics are available from automated alerts, which are both opaque and dichotomous in their output. Presumably, some stratification takes place in the development of the algorithm, but there is no way to know this or with what result. This general practice of making knowledge available through compact conclusions can often be disassembled and broken down in RCTs by examining correlations regarding patient subgroups and effects of sample size, which does allow for some degree of ascertaining the extent to which overall results may be applicable to a particular patient or patient group. The automated production of an AKI e‐alert based on huge amounts of data and modeled using a nontransparent algorithm allows for no such insights into applicability of the outcome to a particular patient nor the direction of any unobserved variable bias.

Instead of supporting decisions, large‐scale adoption of AKI e‐alerts may actually make treatment decisions and assessments more difficult because of the conflation of indicators. The impact on medical knowledge is multifold. *First*, knowledge derived from the automated model about a detected case of AKI only indicates the symptom, most often decreased urine production, and nothing about the underlying cause or the impact of any aggravating co‐morbidities. Instead of increasing our knowledge about this patient, the alert alone, particularly in the hands of a nonspecialist, may actually result in information that leads to harmful treatment decisions, ultimately causing more problems for the patient than a slower assessment made by a trained clinician. *Second*, if the data in each EHR (derived using different metrics and elements of the definition) contribute to the database that drives the output of the algorithmic e‐alert model, the opaque insight that the patient is likely to develop AKI may then be fed back into the database and the “more accurate by way of more data” process perpetuates and increases its uselessness.

## 5 THE DOCTOR–PATIENT RELATIONSHIP: IMPLICATIONS FOR CLINICAL NORMS

The doctor–patient relationship is foundational to clinical practice and adheres to multiple long‐standing norms. Shared decision‐making, informed consent, and moral responsibility are some of the most fundamental aspects of the doctor–patient relationship that are likely to be affected by the operationalization of the definition of clinical conditions into algorithmic models. These aspects of the doctor–patient relationship all involve the delivery of accurate information that is understandable by the patient in a way that allows meaningful participation in decisions about treatment.

Nowhere is this more prominent than in *shared decision‐making*. Shared decision‐making can be viewed as having three pillars: (a) scientific evidence; (b) how the available scientific evidence translates to the clinical condition of the patient; and (c) knowledge about the patient's expectations, goals, preferences, and values. The first two pillars are invariably the domain of physician responsibility. The third is fully within the domain of the patient. Interaction between patient and physician is needed to ensure the patient's expectations are met as much as possible by realistic translation of (theoretical) scientific evidence to the real‐world condition of the patient.^14^ As the medical construct of AKI as defined by KDIGO is muddy, the meaning of the scientific evidence (*first pillar*) for knowledge generation becomes unclear, as AKI might have a different meaning in each study. This also prohibits aggregation of studies into a meta‐analysis, further downplaying the possibility that this research may generate relevant evidence. In this way, the first pillar of scientific evidence loses footing. When AKI is automatically detected by an algorithm, the problem is even further enhanced, as then the physician does not even know what kind of AKI her patient has. The *second pillar* (the patient's condition) also becomes something of a black box because the algorithm has “detected” AKI in a patient, but generates no information about what the source is, which complicating factors may be contributing to the threshold being reached, or even what exactly these thresholds might be in that specific algorithm. For these reasons, the “knowledge” about the patient's condition produced by an automated e‐alert is problematic on many levels and can lead to poor decision‐making by the care team seeking to honor the patient's goals. While the patient in circumstances of an AKI diagnosis in the ICU is rarely in a position to deliberate about treatment alternatives, the possibility for inappropriate interventions deployed with the intent of honoring the patient's previously expressed wishes is increased because of this muddied knowledge about the patient's condition. Because of the opacity of the rationale behind the output, which treatment is likely to produce that result may be more difficult to ascertain. The *third pillar* can remain largely unaffected if there has been an opportunity to confer with the patient prior to acute illness, but the ability of the care team to honor the patient's wishes may be affected by the black box nature of the automation. An automated e‐alert of AKI could, for example, trigger an upgrade of the do‐not‐resuscitate code of a patient who has indicated before that she did not want to have renal replacement therapy. Such a decision would be fair if AKI was defined as “imminent need for renal replacement therapy,” but completely outrageous if AKI was simply defined based on a (temporary and reversible) decrease of UO. This also runs counter to the much discussed ethical AI principles of explainability and transparency.^15^ In the clinical context, the inability to explain the basis for treatment decisions calls many other clinical norms into question.

The *moral responsibility of the attending clinician* is paramount. Both beneficence and nonmalevolence mandate that the physician act responsibly regarding the patient's welfare and well‐being. Advocates of automated detection systems for AKI argue that they require less clinical expertise among staff to produce an AKI alert or diagnosis in a timely manner. This purported benefit is misleading, however. The implementation of AKI alerts is mainly intended to address issues with clinical staff shortages rather than to improve care.

Furthermore, a nonspecialized staff person needing to respond to an AKI e‐alert will know that there is a problem, but will not know what to do about it nor what the risks will be in choosing one course of action over another. As a consequence, either incorrect actions might ensue, or availability of appropriately trained staff may be unnecessarily solicited. The illusion that e‐alerts appear to afford of “being on top” of patient vital signs and “in control,” may actually result in the opposite outcome as a result of understaffing, incorrect allocation of staffing and opaque false positive signaling of life‐threatening conditions.

This points to a moral responsibility to ensure that the patient receives timely and appropriate care in cases of an acute condition developing in the ICU. The extent to which a physician can properly abdicate responsibility for patient monitoring and evaluation to an automated system is de facto limited without a range of complementary systems and adaptations to the current model of care. A nephrologist who leaves nontrained staff to respond to AKI e‐alerts may be violating the two fundamental clinical norms of nonmalevolence and beneficence by both leaving response to a critical condition to personnel who are not able to assess appropriate treatment strategies in a timely manner and by missing the opportunity to intervene effectively in a timely manner, respectively. This suggests a clear need to ensure that the development and translation of criteria like KDIGO are sufficiently transparent so that attending staff can know with some degree of precision what the AKI alert is based on, what factors were not calculated (e.g., UO), and some information about patient characteristics that the model was trained on in order to make assessments about generalizability.

The remaining imprecisions in definitions of medical conditions can be easily handled by the capacity of the human mind for contextualization, but can lead to serious problems when handled by algorithms that completely lack such “common sense.” This is an intrinsic problem that cannot conceivably be solved by requiring greater uniformity in definitions and metrics used to diagnose AKI. The existing semantic confusion and variable implementation of criteria in the available body of evidence moreover prohibit that creating one single sharp definition of AKI would solve this conundrum, as this would imply just another definition for AKI that does not match available evidence. The only way out is to use human expertise and common sense, neither of which are attributes of AI.

## 6 POLICY CONCERNS

The promise of AI in medical treatment and care can only be successfully realized if careful attention is paid to the ways in which medical constructs are operationalized and the manner of embedding them in clinical practice. Failure to attend to predictable consequences arising from both the way in which the model is developed as well as the way in which the applications are used and embedded in clinical practice can lead to a multitude of problems.

One of the concerns about bringing AI into the clinic pertains to *bias and the potential differential impact on patients*. The case of operationalization of KDIGO for algorithmic systems shows how even semantic idiosyncrasies found in medical constructs can contribute to the existence and persistence of health disparities. A seemingly innocent translation of a relatively longstanding set of criteria (in one form or another) when automated and deployed in a growing number of hospitals can ultimately result in *exacerbating health disparities*. Specifically, the culprit here is found, at least partly, in the establishment of the all‐important baseline levels of creatinine. As explained earlier, the different metrics contained in the KDIGO criteria use different methods to arrive at a baseline. For creatinine, the baseline level that informs all subsequent evaluations is determined either by a level determined during pre‐ICU hospital visits (EHR history) or by the level determined upon admission to ICU hospitalization. The systematic application of these two baseline criteria will create two categories of patients—those with frequent access to healthcare and those without.

Access to healthcare in many jurisdictions is dependent on financial resources. People do not seek routine healthcare or nonemergency healthcare if they cannot afford it. Consequently, *poorer patients are less likely to have historical data that can establish their baseline*. Since a diagnosis of AKI is made relative to a baseline, these differing metrics of establishing the baseline can have clinical consequences that result in perpetuation of health disparities. A “physical physician” might notice this disparity and adjust for it, whereas an automated algorithm will simply produce the output AKI or no AKI, but it will not be obvious on which criterion exactly this decision was based. As a consequence, the inequity might remain unnoticed, and the impression will be given that all patients received treatment based on “objective grounds,” whereas in reality, they did not.

The lack of transparency and physician responsibility also have policy ramifications. Shifting the monitoring, evaluation, and detection tasks once performed by trained, experienced clinical staff to an opaque algorithm will necessarily affect *skills of staff* over time. Further, there are concerns about problematic ML models being adopted by clinical staff who are not equipped to assess them.^16^ This conceivably has short and long term implications for the task‐shifting that is expected to accompany AI. The possibility of complementary tasks being taken over by less skilled staff will seem more efficient and economical. Increasing reliance on automated systems will alter what are regarded as clinically necessary skills, and training will be adapted accordingly.

Initially, the meaning of the construct AKI might be different when produced by an AI from what the staff have been taught while in training or what is intended by the term in medical literature, but it will not be transparent for them that there actually *is* a difference and that their response to the AKI should therefore be different. In the long run, new members of staff will simply lack the skills to correct or supplement the AI decisions. The meaning (if any) of the AI output will then only be transparent for the algorithm itself.

Moral responsibility for patient care arguably cannot be shifted to an automated system. For this reason, medical malpractice and medical negligence can still occur with the use of even the most sophisticated technologies. Delegation of moral responsibility in the case of automated alert systems to manufacturers, developers, hospitals adopting the systems, or others in the loop ignores *the moral nature inherent in assuming an obligation for clinical care*. Once this obligation is assumed, for example, by oath, employment, or profession, the moral responsibility to act in ways that will reasonably ensure good clinical care and not be harmful cannot be delegated. This places the responsibility in the hands of clinical staff not only to provide this level of care and caution, but also to only deploy algorithmic systems that facilitate rather than hinder clinical staff's ability to exercise this responsibility.

## 7 CONCLUDING REMARKS

It is a matter of debate whether the solution to the spectrum of dilemmas, diversions, and drawbacks of the semantic black box that is fed into automated algorithmic systems lies in a more refined and transparent translation of KDIGO criteria into the model, adequate regulation and governance, or ensuring appropriate and adequate personnel and processes in the embedding into clinical practice. It is reasonable to suggest that it will take a combination of all of these. While much attention is devoted to the workings of the *technology* as the source of many of the ethical and legal challenges brought about by AI, *communication* still remains at the heart of clinical care. Effective communication is necessary throughout the clinical encounter and increasingly involves technological outputs. Consider the scenario of a patient asking the doctor “Am I going to die?” where the doctor merely responds, “Yes.” The outcome is accurate but tells us so little that we may be inclined to consider it useless or even harmful. While the KDIGO criteria do not set up a scenario quite so extreme, automation of this black box construct compounds an already flawed diagnostic concept that becomes increasingly less useful. When technology interferes with proper understanding and effective communication and hinders the ability to provide reasonable levels of care, then this must be addressed before wide implementation can be considered.

## CONFLICT OF INTEREST

The authors declare no conflict of interest.

## Footnotes

1 Hernán, M. A., Hsu, J., & Healy, B. (2019). A second chance to get causal inference right: A classification of data science tasks. *Chance, 32*(1), 42–49.

2 Kelly, C. J., Karthikesalingam, A., Suleyman, M., Corrado, G., & King, D. (2019). Key challenges for delivering clinical impact with artificial intelligence. *BMC Medicine, 17*(1), 195; Beam, A. L., Manrai, A. K., & Ghassemi, M. (2020). Challenges to the reproducibility of machine learning models in health care. *Journal of American Medical Association, 323*(4), 305–306.

3 Natarajan, P., Frenzel, J., & Smaltz, D. (2017). *Demystifying big data and machine learning for healthcare*. CRC Press, p. 79.

4 Ledley, R. S., & Lusted, L. B. (1959). The use of electronic computers to aid in medical diagnosis. *Proceedings of the IRE, 47*(11), 1970–1977.

5 See for example, Hernán et al., op. cit. note 1; Payne, L. C. (1964). The role of the computer in refining diagnosis. *The Lancet, 284*(7349), 32–35.

6 Lameire, N., Van Biesen, W., & Vanholder, R. (2006). The rise of prevalence and the fall of mortality of patients with acute renal failure: What the analysis of two databases does and does not tell us. *Journal of the American Society of Nephrology, 17*(4), 923–925.

7 Sutherland, S. M., Chawla, L. S., Kane‐Gill, S. L., Hsu, R. K., Kramer, A. A., Goldstein, S. L., Ronco, C., & Bagshaw, S. M., on behalf of the 15 ADQI Consensus Group. (2016). Utilizing electronic health records to predict acute kidney injury risk and outcomes: Workgroup statements from the 15th ADQI Consensus Conference. *Canadian Journal of Kidney Health and Disease, 3*, 11; Sutherland, S. M., Goldstein, S. L., & Bagshaw, S. M. (2018). Acute kidney injury and big data. In X. Ding, M. H. Rosner, & C. Ronco (Eds.), *Acute kidney injury ‐ Basic research and clinical practice* (Vol. 193, pp. 55–67). Karger Publishers.

8 Park, S., Baek, S. H., Ahn, S., Lee, K. H., Hwang, H., Ryu, J., Ahn, S. Y., Chin, H. J., Na, K. Y., Chae, D.‐W., & Kim, S. (2018). Impact of electronic acute kidney injury (AKI) alerts with automated nephrologist consultation on detection and severity of AKI: A quality improvement study. *American Journal of Kidney Diseases, 71*(1), 9–19; Kate, R. J., Perez, R. M., Mazumdar, D., Pasupathy, K. S., & Nilakantan, V. (2016). Prediction and detection models for acute kidney injury in hospitalized older adults. *BMC Medical Informatics and Decision Making, 16*(1), 39.

9 Van Biesen, W., Vanmassenhove, J., & Decruyenaere, J. (2020). Prediction of acute kidney injury using artificial intelligence: Are we there yet? *Nephrology, Dialysis, Transplantation, 35*(2), 204–205.

10 Lameire et al., op. cit. note 6.

11 Van Acker, P., Van Biesen, W., Nagler, E., Koobasi, M., Veys, N., & Van Massenhove, J. (2021). Risk prediction models for acute kidney injury in adults: An overview of systematic reviews. *PLoSOne, 16*(4), e0248899. https://doi.org/10.1371/journal.pone.0248899

12 Khwaja, A. (2012). KDIGO clinical practice guidelines for acute kidney injury. *Nephron Clinical Practice, 120*(4), c179–c184.

13 Gameiro, J., Agapito Fonseca, J., Jorge, S., & Lopes, J. A. (2018). Acute kidney injury definition and diagnosis: A narrative review. *Journal of Clinical Medicine, 7*(10), 307.

14 See for example, Brock, D. W. (1991). The ideal of shared decision making between physicians and patients. *Kennedy Institute of Ethics Journal, 1*(1), 28–47.

15 See European Commission. (2020). *White paper on artificial intelligence – A European approach to excellence and trust*. European Commission.

16 Wilkinson, J., Arnold, K. F., Murray, E. J., van Smeden, M., Carr, K., Sippy, R., de Kamps, M., Beam, A., Konigorski, S., Lippert, C., Gilthorpe, M. S., & Tennant, P. W. (2020). Time to reality check the promises of machine learning‐powered precision medicine. *The Lancet Digital Health, 2*(12), E677–E680.

